# A study on the complex interplay between inflammation and severe mental disorders (SMInflam)

**DOI:** 10.1192/j.eurpsy.2024.1423

**Published:** 2024-08-27

**Authors:** B. Della Rocca, M. Luciano, G. De Felice, M. Di Vincenzo, C. Toni, E. Arsenio, G. Sampogna, A. Fiorillo

**Affiliations:** Università della Campania “L. Vanvitelli”, Naples, Italy

## Abstract

**Introduction:**

An alteration of inflammatory indices has been reported in several major mental disorders. This alteration seems to be related to disease severity and treatment resistance, but its pathophysiological meaning remains to be established. Patients with severe mental disorders tend to have increased levels of circulating cytokines and increased microglial activity in the central nervous system, suggesting that inflammation may contribute to the onset, or chronicity, of mental disorders. Detecting inflammation‐relevant symptom clusters across mental disorders may represent an important step towards precision medicine in psychiatry.

**Objectives:**

The SMInflam project is a longitudinal, observational, real-world study which aims to: assess a set of inflammatory indices at baseline in a sample of patients with the diagnosis of a major mental disorder; identify inflammatory profiles of these patients using a latent class analysis approach; assess the response to pharmacological treatments of patients with different inflammatory profiles; re-assess the inflammatory indices and profiles at several times during follow-up and test their correlation with the evolution of psychopathology.

**Methods:**

The sample will consist of 50 patients with a diagnosis of a major mental disorders consecutively enrolled at the outpatient unit of the Department of Psychiatry of University of Campania. All enrolled patients will be administered a set of reliable and validated psychopathological assessment tools. We will perform a complete physical evaluation, and a battery of laboratory tests. Peripheral markers of chronic inflammation will be assessed. Clinical and biological assessments will be performed at baseline (T0) and after 3 and 6 months (respectively, T1 and T2).

**Results:**

Expected results include the evaluation of the levels of inflammatory indices in a varied sample of patients with severe mental disorders. According to the pre-post design, these aspects will be evaluated before the start and at the follow-up. We will also take into consideration the role of confounding factors such as age and gender, which represent a critical biological variable influencing such inflammatory pathways.

**Conclusions:**

Collected data will be used for having a more informative, reliable and valid characterization of psychopathology in a vast sample of patients with severe mental disorders. Our study may represent the first of a new wave of methodologically-sound studies on the role of inflammation and psychopathology in patients with severe mental disorders.

**Disclosure of Interest:**

None Declared

